# Plant essential oils synergize various pyrethroid insecticides and antagonize malathion in *Aedes aegypti*

**DOI:** 10.1111/mve.12380

**Published:** 2019-05-17

**Authors:** E. J. Norris, A. D. Gross, L. C. Bartholomay, J. R. Coats

**Affiliations:** ^1^ Department of Entomology Iowa State University Ames IA U.S.A.; ^2^ Department of Entomology Virginia Polytechnic Institute and State University Blacksburg VA U.S.A.; ^3^ Department of Pathobiological Sciences University of Wisconsin Madison WI U.S.A.

**Keywords:** pyrethroids, plant essential oils, enhance, synergize, malathion

## Abstract

Pyrethroid resistance is a significant threat to agricultural, urban and public health pest control activities. Because economic incentives for the production of novel active ingredients for the control of public health pests are lacking, this field is particularly affected by the potential failure of pyrethroid‐based insecticides brought about by increasing pyrethroid resistance. As a result, innovative approaches are desperately needed to overcome insecticide resistance, particularly in mosquitoes that transmit deadly and debilitating pathogens. Numerous studies have demonstrated the potential of plant essential oils to enhance the efficacy of pyrethroids. The toxicity of pyrethroids combined with plant oils is significantly greater than the baseline toxicity of either oils or pyrethroids applied alone, which suggests there are synergistic interactions between components of these mixtures. The present study examined the potential of eight plant essential oils applied in one of two concentrations (1% and 5%) to enhance the toxicity of various pyrethroids (permethrin, natural pyrethrins, deltamethrin and β‐cyfluthrin). The various plant essential oils enhanced the pyrethroids to differing degrees. The levels of enhancement provided by combinations of plant essential oils and pyrethroids in comparison with pyrethroids alone were calculated and synergistic outcomes characterized. Numerous plant essential oils significantly synergized a variety of pyrethroids; type I pyrethroids were synergized to a greater degree than type II pyrethroids. Eight plant essential oils significantly enhanced 24‐h mortality rates provided by permethrin and six plant essential oils enhanced 24‐h mortality rates obtained with natural pyrethrins. By contrast, only three plant essential plants significantly enhanced the toxicity of deltamethrin and β‐cyfluthrin. Of the plant essential oils that enhanced the toxicity of these pyrethroids, some produced varying levels of synergism and antagonism. Geranium, patchouli and Texas cedarwood oils produced the highest levels of synergism, displaying co‐toxicity factors of > 100 in some combinations. To assess the levels of enhancement and synergism of other classes of insecticide, malathion was also applied in combination with the plant oils. Significant antagonism was provided by a majority of the plant essential oils applied in combination with this insecticide, which suggests that plant essential oils may act to inhibit the oxidative activation processes within exposed adult mosquitoes.

## Graphical abstract

- Plant essential oils enhance the efficacy of a variety of pyrethroids; higher levels of enhancement occur when oils are applied in combination with type I pyrethroids compared with type II pyrethroids.
- Plant essential oils synergize specific pyrethroids; higher levels of synergism occur when oils are applied in combination with type I pyrethroids.
- Plant essential oils antagonize malathion toxicity against *Aedes aegypti*.

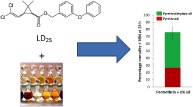

## Introduction

Synergists are important components of some insecticide formulations and prevent the detoxification of active ingredients of insecticides (Metcalf, 1967; Yu, 2015). These compounds function by inhibiting the enzymatic pathways that broadly metabolize and aid in the excretion of particular toxicants encountered by insects within their environments (Metcalf, 1967). When used properly, synergists increase the overall toxicity of insecticidal active ingredients by increasing the bioavailability of the active compounds to interact with the target in the insect. Moreover, synergists can be utilized to reclaim the efficacy of certain insecticidal chemicals to which some insect populations have become resistant (Darriet & Chandre, 2011; Somwang *et al*., 2011; Gao *et al*., 2012). If insecticide resistance to select modes of action is mediated by upregulated detoxification mechanisms, synergists may inhibit these processes. In this scenario, synergists may facilitate similar insecticidal activity in both insecticide‐resistant and insecticide‐susceptible strains of mosquito.

Plant essential oils and their constituents represent promising additives to insecticides. Numerous studies have demonstrated that plant terpenoids act at a variety of molecular targets (Miyazawa & Kameoka, 1997; Mills *et al*., 2004; Lopez & Pascual‐Villalobos, 2010; Tong & Coats, 2010; Anderson & Coats, 2012; Gross *et al*., 2017b). Many of these targets are distinct from mammalian targets and therefore these constituents should be safer for humans and non‐target animals (Isman, 2000). Furthermore, because of their novel mechanisms of action, there is little chance of the development of cross‐resistance with compounds currently available on the market. Moreover, multiple studies have demonstrated the ability of plant essential oils to enhance the efficacy of different insecticidal chemistries (Joffe *et al*., 2012; Gross *et al*. 2017a). Although this mechanism has not been fully characterized, the present authors hypothesize that this enhancement is caused, in part, by the inhibition of the degradation of these insecticides by detoxification enzymes. As the development of new insecticidal active ingredients is costly and prohibitive (Sparks & Lorsbach, 2017), plant essential oils may represent a relatively cost‐effective means of increasing the efficacy of various insecticidal formulations. Moreover, many of these chemistries are recognized as possessing low levels of toxicity to mammals and other non‐target organisms, and are not likely to be persistent in the environment (Isman, 2000).

Because of the relatively small size of the mosquito control industry and the importance of mosquito control in countries with scant economic resources, there is little economic incentive to create new insecticidal technologies (Hemingway *et al*., 2006; Knapp *et al*., 2015). Instead, mosquito control research and development rely, in large, on the repurposing of agricultural pest control chemistries (Mnzava *et al*., 2015). Of the wide variety of agrochemicals available in agricultural pest control, only select chemistries are ideal for mosquito control. Currently, only four classes of insecticide are available for the control of mosquitoes. The persistence of synthetic insecticides in the environment and the toxicity of these chemistries to mammals and non‐target organisms are important characteristics that limit the viability of mosquito control insecticides. Novel chemistries for the control of public health vector insect species must also result in rapid knock‐down in the target species, particularly in the context of active pathogen transmission, and must rapidly intervene and kill adult mosquitoes (Nauen, 2007). Moreover, the relatively few available and approved mosquito control chemistries are beginning to fail in the field as wild mosquito populations continue to become resistant to these classes of insecticide.

Gross *et al*. (2017a) demonstrated that many essential oils enhance permethrin efficacy to a higher degree than piperonyl butoxide (PBO) in two species of mosquito. Moreover, according to the findings of previous work (Norris *et al*., 2015), the baseline toxicity levels of these oils are not sufficient to completely account for the enhanced efficacy of mixtures of plant essential oils and synthetic insecticides against adult female mosquitoes. The present study explored the potential of plant essential oils to enhance the insecticidal effects of diverse synthetic pyrethroids and natural pyrethrins. Plant essential oils were applied in combination with a pro‐insecticide, malathion, which requires oxidative activation to exert a toxic effect (Wickham, 1998), to test whether plant essential oils inhibit the oxidative activation of this insecticide.

## Materials and methods

### *Insect rearing*

Adult female *Aedes aegypti* (Liverpool strain) (Diptera: Culicidae) were reared according to standard laboratory procedures maintained by the Medical Entomology Laboratory at Iowa State University. In short, mosquito colonies were maintained at a constant temperature of 30 °C and relative humidity (RH) of 80 ± 10% and provided with 10% sucrose water pads *ad libitum*. Colony cages were provided with weekly bloodmeals of defibrinated sheep blood (Hemostat Laboratories, Inc., Dixon, CA, U.S.A.) to promote the development of eggs. Eggs were collected on paper towels once per week and deposited into aluminium pans for hatching. Larvae were fed TetraMin® fish flakes (Tetra, Inc., Blacksburg, VA, U.S.A.) every other day and larval water was replaced twice per week. Pupae were collected and subsequently separated based on size. Female pupae were collected and kept in 12‐oz (355‐mL) deli cartons, at a density of approximately 50 females per carton. Unmated adult female mosquitoes were used at 2–5 days post‐eclosion in all studies.

### *Topical application of insecticide and insecticide–plant essential oil mixtures*

The technical grade synthetic insecticides and natural pyrethrins used for this study were obtained from a variety of sources. Permethrin Z:E 40:60 and natural pyrethrins (20% pure in ethanol) were obtained from EcoSMART Technologies, Inc. (Roswell, GA, U.S.A.). Deltamethrin, β‐cyfluthrin and malathion were obtained from Sigma Aldrich Corp. (St Louis, MO, U.S.A.). To obtain dose–response data for each insecticide, varying concentrations of synthetic insecticides were applied to individual adult female mosquitoes in a volume of 0.2 μL per mosquito. Plant essential oils were obtained from Berje, Inc. (Carteret, NJ, U.S.A.). All insecticidal active ingredients and mixtures were applied in acetone throughout this study. Treated mosquitoes were kept in an environmental chamber at a constant temperature of 28 °C and RH 80 ± 10%. Concentrations that caused between 5% and 95% mortality at 24 h after the initial exposure were chosen. Gas chromatography–mass spectrometry data for the oils included in this study are provided in Table S1.

After LD_25_ (lethal dose required to produce 25% mortality) values had been calculated for each of the synthetic insecticides, mixtures of a discrete dose of synthetic insecticide, which corresponded to the LD_25_, and either 1% or 5% plant essential oil by weight were made. This facilitated the screening of plant essential oil mixtures at both low and high doses. Because enhancement by plant essential oils may be mediated by either synergistic processes (e.g. enhancement of cuticular penetration, inhibition of monooxygenase detoxification of synthetic insecticides, etc.) or additive toxicity, both low and high plant essential oil dosage mixtures were screened to assess the effects of plant terpenoids applied in combination with synthetic and natural insecticides.

### *Data analysis*

*LD*_*50*_ *and LD*_*25*_ *value characterization*. The lethal dosages required to produce 25% mortality (LD_25_) and 50% mortality (LD_50_) at 24 h were calculated in sas Version 9.4 (SAS Institute, Inc., Cary, NC, U.S.A.) using a proc probit model with OPTC correction. At least five concentrations of each insecticide producing mortality rates of 5–95% at 24 h were chosen. A minimum of three biological replicates were used for theoretical dose–response calculation to ensure accurate characterizations of synthetic insecticide toxicity. A minimum of at least 750 *Ae. aegypti* mosquitoes were used for each insecticide to guarantee the reproducibility of these studies. When χ^2^‐values were high (*P* < 0.05), a heterogeneity co‐factor was applied to the data to provide for a better fit of the data (probit, sas Version 9.4).

### *Oil‐alone, insecticide‐alone and mixture toxicity studies*

For each combination of plant essential oil and insecticide, a minimum of three replicates were performed with *Ae. aegypti* using two concentrations of oil (1% and 5% w/v) paired with the LD_25_ of each insecticide. To assess synergism by plant essential oils in each mixture, mosquitoes were also exposed to 0.2 μL plant essential oils alone at both the 1% and 5% w/v concentrations. For this study, patchouli, *Origanum* (i.e. Oregano), cedarwood [Texas type (CWT)], clove leaf, clove bud, cedarwood [Moroccan type (CWM)], basil and cinnamon bark oils were used. For each plant essential oil and insecticide mixture at both the 1% and 5% plant essential oil concentrations, three replicates of, respectively, a 1% oil alone, 5% oil alone and an LD_25_ of each insecticide application were also performed. This strategy was intended to account for the percentage effects of each of these components (i.e. LD_25_ insecticide alone, 1% oil alone, 5% oil alone, 1% oil + LD_25_ insecticide, 5% oil + LD_25_ insecticide) across biological replicates. This form of analysis takes into account any minor changes in effect among different biological cohorts and prevents cohort bias in the analysis. To assess statistical differences among the 24‐h mortality rates provided by 1% and 5% plant essential oil + LD_25_ insecticide compared with the LD_25_ insecticide applied alone, a Student's *t*‐test was performed (α = 0.05) to assess changes in toxicity that occurred with the addition of the plant essential oils compared with the insecticide alone. Piperonyl butoxide was used throughout this study as a commercially used pyrethroid synergist.

### *Percentage enhancement*

Toxicity was calculated as a percentage of the average mortality of *Ae. aegypti* exposed to each plant essential oil and synthetic insecticide or natural pyrethrin mixture. Percentage enhancement was calculated using a method similar to that used by Gross *et al*. (2017a) relying on the following equation for each replicate:

$$\begin{array}{ll} & \text{Percentage Enhancement}\\ & =\frac{\begin{array}{l} \left(\text{toxicity of mixture}\right)-(\text{toxicity of synthetic}\\ \text{insecticide or natural pyrethrins}) \end{array}}{\left(\text{toxicity of synthetic insecticide or natural pyrethrins}\right)}\\ & \times 100 \end{array}$$

A Student's *t*‐test (α = 0.05) was performed to evaluate whether the percentage enhancement caused by each plant essential oil was statistically significant compared with PBO. All statistical analyses were performed using sas Version 9.4.

### *Synergism calculations*

The synergy of various components with the plant essential oil and insecticidal mixtures was characterized using a previously established method (Mansour *et al*., 1966). In short, the co‐toxicity factor was calculated using the equation:

$$\begin{array}{ll} & \mathrm{Co}-\text{toxicity factor}\\ & =\frac{\left(\text{observed}\%\text{mortality}\right)-\left(\text{expected}\%\text{mortality}\right)}{\left(\text{expected}\%\text{mortality}\right)}\\ & \times 100 \end{array}$$

with expected mortality calculated as the sum of the percentage mortalities achieved by, respectively, the pyrethroid alone and the plant essential oil. The co‐toxicity factor can be used to assess whether an antagonistic, additive or synergistic relationship exists among components within a mixture compared with the individual components. Values lower than − 20 suggest an antagonistic relationship, values between − 20 and 20 suggest an additive character, and values greater than 20 suggest a synergistic character. Values of less than − 15 but greater than − 20 or greater than 15 but less than 20 were considered to indicate trends towards antagonistic and synergistic characters, respectively.

## Results

The LD_25_ and LD_50_ values for *Ae. aegypti* exposed to each insecticide were calculated [Table 1; permethrin data published previously in Norris *et al*. (2015)]. The most toxic insecticide for *Ae. aegypti* in this study was β‐cyfluthrin, with an estimated LD_50_ value of 0.03 μg/g mosquito. This was followed by permethrin, deltamethrin, malathion and natural pyrethrins, with LD_50_ values of 0.42 μg/g, 0.58 μg/g, 1.42 μg/g and 6.21 μg/g mosquito, respectively. The LD_25_ values for β‐cyfluthrin, deltamethrin, permethrin, malathion and natural pyrethrins were 0.004 μg/g, 0.01 μg/g, 0.19 μg/g, 1.02 μg/g and 2.87 μg/g mosquito, respectively. A 207‐fold range in LD_50_ values and a 718‐fold range in LD_25_ values from the least toxic to most toxic insecticide screened were observed.

**Table 1:** Dose–response data for *Aedes aegypti* topically exposed to a panel of insecticides.

Insecticide	*n*	LD_25_ (μg/g mosq.)	LD_50_ (μg/g mosq.)	Slope (SE)	95% CI (of LD_50_)	χ^2^‐value (d.f.)
Permethrin	1300	0.19	0.42	1.93 (0.3)	0.3–0.5	129.6 (43)
Natural pyrethrins	850	2.87	6.21	2.015 (0.3)	4.5–8.6	98.5 (27)
β‐cyfluthrin	1575	0.004	0.03	0.84 (0.15)	0.02–0.06	199.6 (51)
Deltamethrin	1525	0.01	0.58	0.39 (0.08)	0.2–5.2	120.0 (50)
Malathion	900	1.02	1.42	4.73 (0.73)	1.3–1.6	92.3 (28)

CI, confidence interval; SE, standard error.

### *Combinations with permethrin*

**Figure 1:**
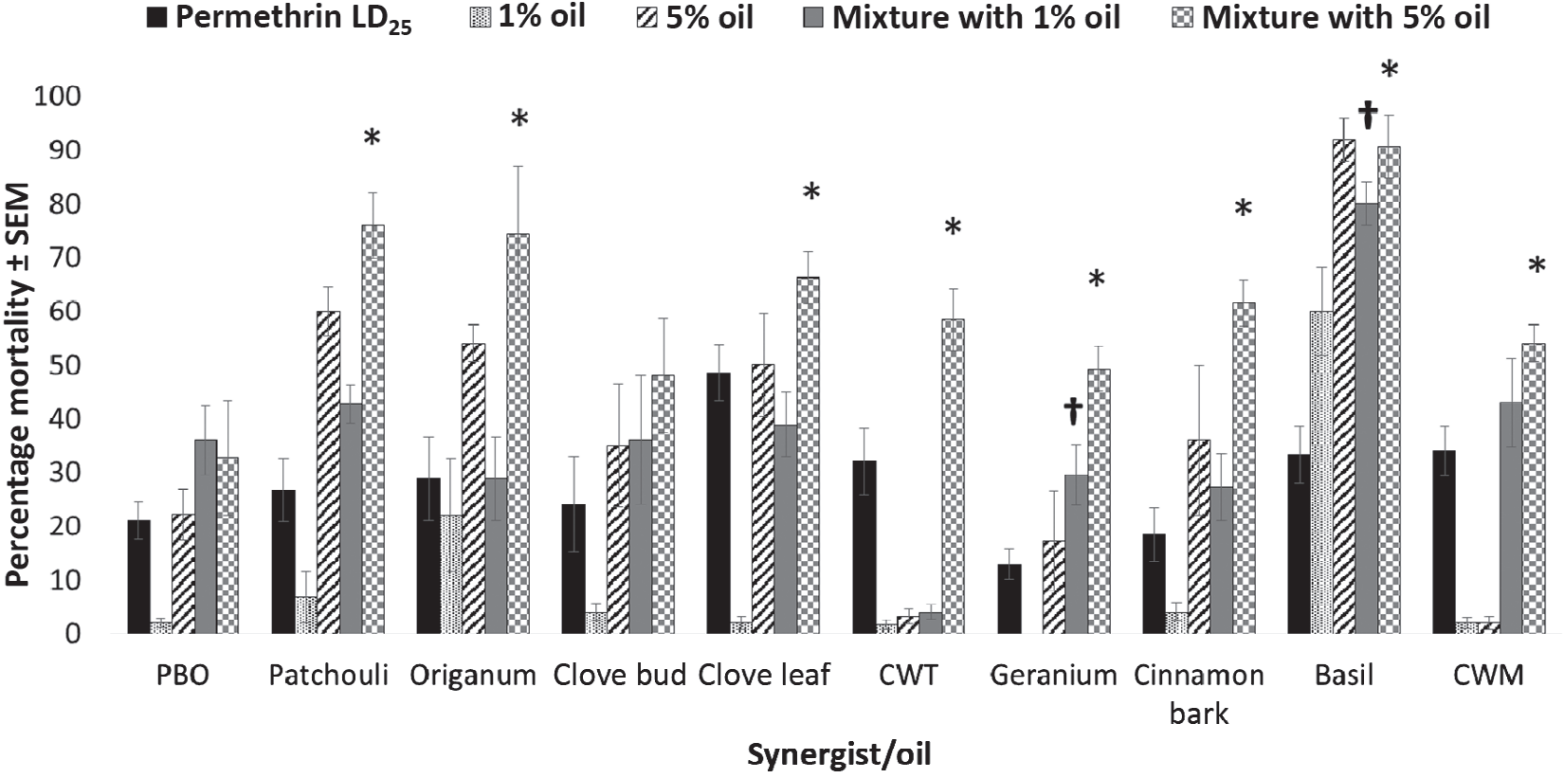
Percentage mortality in adult female *Aedes aegypti* at 24 h after application of each synergist or plant essential oil in combination with permethrin. *, statistically significant difference in percentage mortality caused by the 5% synergist/plant essential oil + LD_25_ permethrin combination compared with the LD_25_ permethrin‐alone application. †, statistically significant difference in percentage mortality caused by the 1% synergist/plant essential oil + LD_25_ permethrin combination compared with the LD_25_ permethrin‐alone application. PBO, piperonyl butoxide; CWT, Texas cedarwood; CWM, Moroccan cedarwood.

Mortality rates in *Ae. aegypti* induced by exposure to mixtures of plant essential oils and permethrin are shown in Fig. 1. The 24‐h percentage mortality caused by the LD_25_ of permethrin alone ranged from 12.8 ± 3.0% to 48.6 ± 5.0% in trials associated with each plant essential oil. The plant essential oil most toxic to *Ae. aegypti* in this experiment was basil, which provided mortality rates of 60.0 ± 8.0% and 92.0 ± 4.0% in the 1% and 5% oil‐alone challenges. The oil least toxic to *Ae. aegypti* was CWT, which provided mortality rates of 1.6 ± 1.0% and 3.2 ± 1.0% at 24 h in the oil‐alone challenges. Among the LD_25_ permethrin + 1% plant essential oil exposures, a significant increase in mortality compared with that of the LD_25_ of permethrin applied alone was observed for both geranium (*P* = 0.03) and basil (*P* = 0.0022) oils. In the LD_25_ permethrin + 5% plant essential oil exposures, multiple oils produced significant increases in *Ae. aegypti* mortality compared with the LD_25_ permethrin alone exposure. Patchouli (*P* = 0.042), *Origanum* (*P* = 0.015), clove leaf (*P* = 0.0279), CWT (*P* = 0.0047), geranium (*P* = 0.003), cinnamon bark (*P* = 0.0002), basil (*P* = 0.0019) and CWM (*P* = 0.0145) oils all produced mortality in *Ae. aegypti* that was higher than that effected by the LD_25_ of permethrin alone. Piperonyl butoxide did not significantly increase the toxicity of permethrin at either the 1% or 5% levels applied in combination with permethrin. Numerous plant essential oils and PBO also caused significant synergism when applied to *Ae. aegypti* in combination with permethrin (Table 2). Piperonyl butoxide, patchouli, clove bud, geranium and cinnamon bark oils caused significant synergism when applied to *Ae. aegypti* in combination with permethrin at the 1% concentration. Three plant essential oils caused significant synergism when applied in combination with permethrin at the 5% level: CWT, cinnamon bark and CWM oils all caused significant synergism of this pyrethroid. By contrast, some oils caused significant antagonism. Although it was synergistic when applied in combination with permethrin at the 1% level, PBO was antagonistic at the 5% level. *Origanum*, clove leaf and CWT oils were all antagonistic when applied at the 1% level, and clove leaf and basil oils were antagonistic at the 5% level in combination with permethrin.

**Table 2:** Synergism and antagonism of an LD_25_ of permethrin by piperonyl butoxide (PBO) and select plant essential oils applied to *Aedes aegypti* adult females. Values lower than − 20 suggest an antagonistic relationship, values between − 20 and 20 suggest an additive character, and values greater than 20 suggest a synergistic character.

	Oil/synergist 1%					Oil/synergist 5%				
	Mortality, %					Mortality, %				
Synergist/plant oil	Permethrin	Synergist/oil alone	Expected	Observed	Co‐toxicity factor	Permethrin	Synergist/oil alone	Expected	Observed	Co‐toxicity factor
PBO	21.1	2.0	23.1	36.0	55.8	21.1	22.2	43.3	32.7	−24.5
Patchouli	26.7	6.7	33.4	42.7	27.8	26.7	60.0	86.7	76.0	−12.3
Origanum	28.8	22.0	50.8	28.8	−43.3	28.8	54.0	82.8	74.4	−10.1
Clove bud	24.0	4.0	28.0	36.0	28.6	24.0	35.0	59.0	48.0	−18.6
Clove leaf	48.6	2.0	50.6	38.9	−23.1	48.6	50.0	98.6	66.3	−32.8
Texas cedarwood	32.0	1.6	33.6	4.0	−88.1	32.0	3.2	35.2	58.4	65.9
Geranium	12.8	0.0	12.8	29.6	131.3	12.8	17.3	30.1	49.2	63.5
Cinnamon bark	18.4	4.0	22.4	27.2	21.4	18.4	36.0	54.4	61.6	13.2
Basil	33.3	60.0	93.3	80.0	−14.3	33.3	92.0	125.3	90.6	−27.7
Moroccan cedarwood	34.0	2.0	36.0	43.0	19.4	34.0	2.0	36.0	54.0	50.0

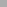
Light grey shading refers to synergistic co‐toxicity factors.

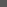
Dark grey shading refers to antagonistic co‐toxicity factors

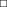
No shading corresponds to additive co‐toxicity factors.

### *Combinations with natural pyrethrins*

**Figure 2:**
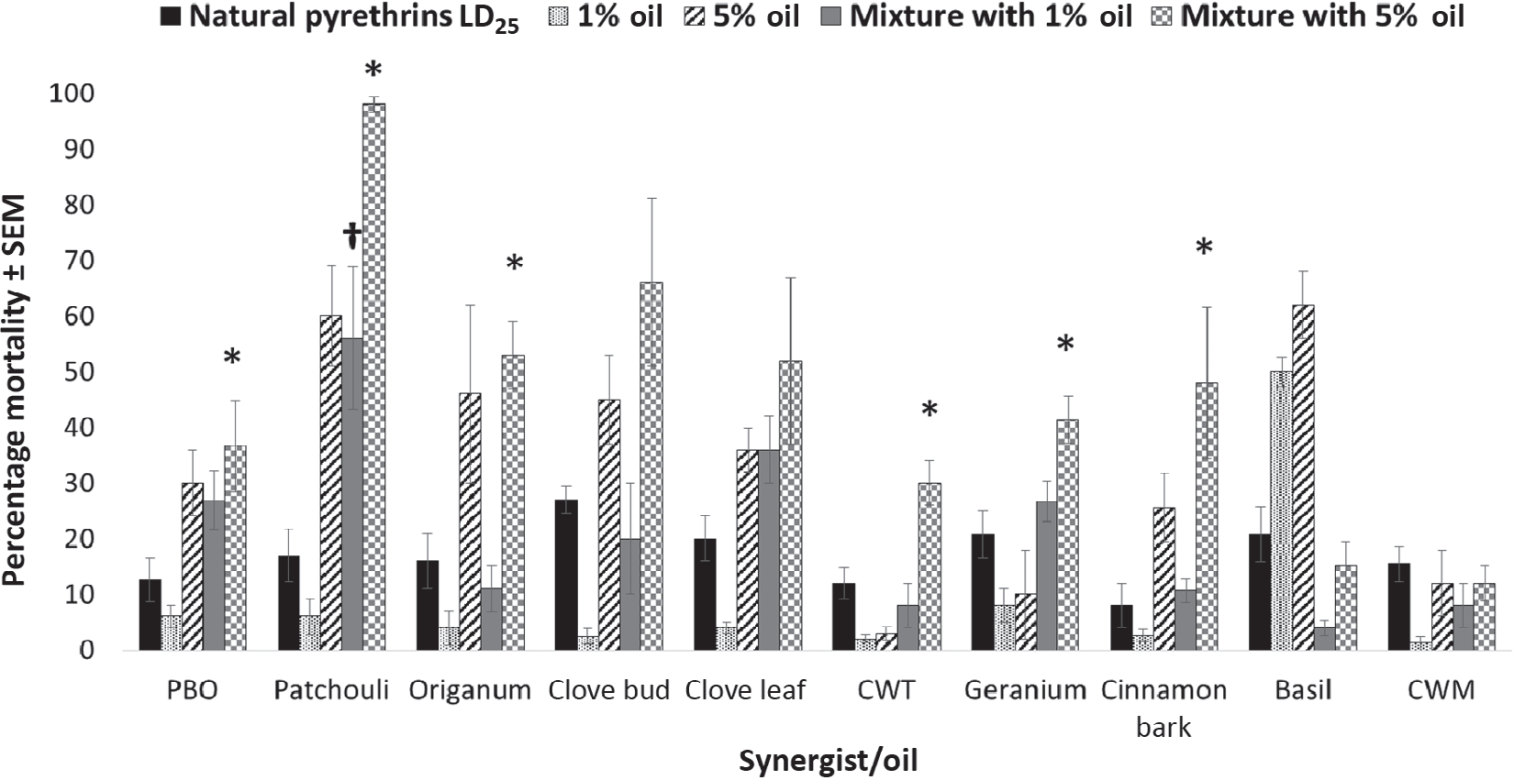
Percentage mortality in adult female *Aedes aegypti* at 24 h after application of each synergist or plant essential oil in combination with natural pyrethrins. *, statistically significant difference in percentage mortality caused by the 5% synergist/plant essential oil + LD_25_ natural pyrethrins combination compared with the LD_25_ natural pyrethrins‐alone application. PBO, piperonyl butoxide; CWT, Texas cedarwood; CWM, Moroccan cedarwood.

A different trend was observed in the effects on *Ae. aegypti* of plant essential oil combinations with natural pyrethrins (Fig. 2). Again, levels of plant essential oil toxicity to *Ae. aegypti* observed in the natural pyrethrins trial were similar to those in the previous experiments with permethrin, with CWT being the least toxic and basil the most toxic plant essential oil when applied alone at the 1% and 5% concentrations. Moreover, the LD_25_ of natural pyrethrins produced mortality in *Ae. aegypti* with low variability ranging from 8.0 ± 4.0% to 24.7 ± 2.0% in all plant essential oil trials. Although numerical increases in 24‐h mortality were observed for many oils applied in combination with the LD_25_ of natural pyrethrins, only patchouli oil (*P* = 0.0288) produced a significant increase in mortality in *Ae. aegypti* at the 1% level when applied in tandem with natural pyrethrins. Piperonyl butoxide was not capable of enhancing the mortality caused by natural pyrethrins at this level. Multiple plant essential oils (applied at 5%) significantly increased 24‐h mortality in *Ae. aegypti* over the LD_25_ of natural pyrethrins alone. The essential oils were those of patchouli (*P* = 0.0255), *Origanum* (*P* = 0.0032), CWT (*P* = 0.0074), geranium (*P* = 0.0147) and cinnamon bark (*P* = 0.042), which caused mortality rates of, respectively, 98.0 ± 1.0%, 53.0 ± 6.0%, 30.0 ± 4.0%, 41.3 ± 4.0% and 48.0 ± 13.5% at 24 h. Although combinations of other oils with natural pyrethrins induced numerically higher mortality in *Ae. aegypti* than some of these oils, the differences were not statistically significant. Significant synergism was also noted for plant essential oils applied to *Ae. aegypti* in combination with natural pyrethrins (Table 3). Piperonyl butoxide, patchouli oil and clove leaf oil all produced significant synergism when applied at the 1% level in combination with natural pyrethrins. Patchouli, CWT, geranium and cinnamon bark oils all produced significant synergism when applied at the 5% level in combination with natural pyrethrins. Again, numerous oils produced significant levels of antagonism when applied at either of these two concentrations in combination with natural pyrethrins.

**Table 3:** Synergism and antagonism of an LD_25_ of natural pyrethrins by piperonyl butoxide (PBO) and select plant essential oils for *Aedes aegypti* at various concentrations. Values lower than − 20 suggest an antagonistic relationship, values between − 20 and 20 suggest an additive character, and values greater than 20 suggest a synergistic character.

	Oil/synergist 1%					Oil/synergist 5%				
	Mortality, %					Mortality, %				
Synergist/plant oil	Natural pyrethrins	Synergist/oil alone	Expected	Observed	Co‐toxicity factor	Natural pyrethrins	Synergist/oil alone	Expected	Observed	Co‐toxicity factor
PBO	12.6	6.0	18.6	26.9	44.6	12.6	30.0	42.6	36.6	−14.1
Patchouli	17.0	6.0	23.0	56.0	143.5	17.0	60.0	77.0	98.0	27.3
Origanum	16.0	4.0	20.0	11.0	−45.0	16.0	46.0	62.0	53.0	−14.5
Clove bud	27.0	2.5	29.5	20.0	−32.2	27.0	45.0	72.0	66.0	−8.3
Clove leaf	20.0	4.0	24.0	36.0	50.0	20.0	36.0	56.0	52.0	−7.1
Texas cedarwood	12.0	2.0	14.0	8.0	−42.9	12.0	3.0	15.0	30.0	100.0
Geranium	20.7	8.0	28.7	26.7	−7.0	20.7	10.0	30.7	41.3	34.5
Cinnamon bark	8.0	2.6	10.6	10.7	0.9	8.0	25.6	33.6	48.0	42.9
Basil	20.8	50.0	70.8	4.0	−94.4	20.8	62.0	82.8	15.2	−81.6
Moroccan cedarwood	15.5	6.0	21.5	8.0	−62.8	15.5	12.0	27.5	12.0	−56.4

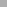
Light grey shading refers to synergistic co‐toxicity factors.

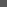
Dark grey shading refers to antagonistic co‐toxicity factors.

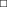
No shading corresponds to additive co‐toxicity factors.

### *Combinations with deltamethrin*

**Figure 3:**
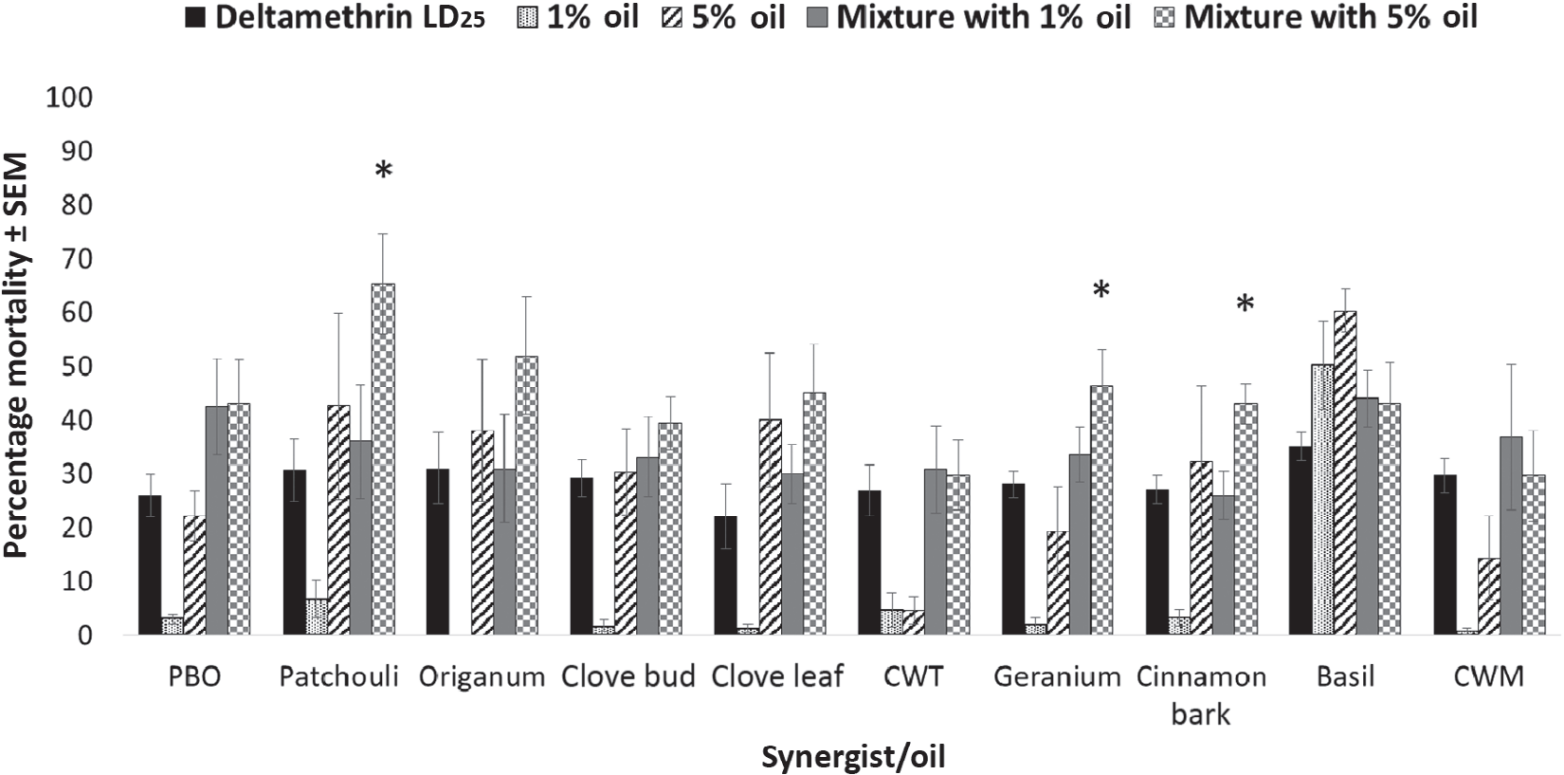
Percentage mortality in adult female *Aedes aegypti* at 24 h after application of each synergist or plant essential oil in combination with deltamethrin. *, statistically significant difference in percentage mortality caused by the 5% synergist/plant essential oil + LD_25_ deltamethrin combination compared with the LD_25_ deltamethrin‐alone application. PBO, piperonyl butoxide; CWT, Texas cedarwood; CWM, Moroccan cedarwood.

When applied in combination with the LD_25_ of deltamethrin, select plant essential oils produced higher 24‐h mortality in *Ae. aegypti* than the LD_25_ of deltamethrin alone (Fig. 3). *Aedes aegypti* mortality after exposure to the LD_25_ of deltamethrin ranged from 22.0 ± 6.0% to 35.0 ± 2.7% across all plant essential oil challenges. No plant essential oil caused a significant increase in mortality in *Ae. aegypti* at 1% in combination with an LD_25_ of deltamethrin. Three plant essential oils [patchouli (*P* = 0.0342), geranium (*P* = 0.011) and cinnamon bark (*P* = 0.027)] at 5% and in combination with an LD_25_ of deltamethrin caused significant increases in mortality in *Ae. aegypti* compared with deltamethrin applied alone. Mortality rates of 65.3 ± 9.3%, 46.4 ± 6.5% and 43.0 ± 3.7% were observed in *Ae. aegypti* exposed to mixtures of the LD_25_ of deltamethrin with patchouli, geranium and cinnamon bark oils, respectively. Although some plant essential oils caused significant synergism of type II pyrethroids, significantly less synergism was noted for these pyrethroids compared with type I pyrethroids (Tables 3 and 4). In summary, only two oils caused significant synergism of deltamethrin. Piperonyl butoxide, clove leaf oil and CWM oil all caused significant synergism of deltamethrin and only when applied at the 1% concentration.

**Table 4:** Synergism and antagonism of an LD_25_ of deltamethrin by piperonyl butoxide (PBO) and select plant essential oils at various concentrations applied to *Aedes aegypti*. Values lower than − 20 suggest an antagonistic relationship, values between − 20 and 20 suggest an additive character, and values greater than 20 suggest a synergistic character.

	Oil/synergist 1%					Oil/synergist 5%				
	Mortality, %					Mortality, %				
Synergist/plant oil	Deltamethrin	Synergist/oil alone	Expected	Observed	Co‐toxicity factor	Deltamethrin	Synergist/oil alone	Expected	Observed	Co‐toxicity factor
PBO	26.0	3.0	29.0	42.5	46.6	26.0	22.2	48.2	43.0	−10.8
Patchouli	30.7	6.7	37.4	36.0	−3.7	30.7	42.6	73.3	65.3	−10.9
Origanum	31.0	0.0	31.0	31.0	0.0	31.0	38.0	69.0	52.0	−24.6
Clove bud	29.1	1.7	30.8	33.1	7.5	29.1	30.3	59.4	39.4	−33.7
Clove leaf	22.0	1.2	23.2	30.0	29.3	22.0	40.0	62.0	45.0	−27.4
Texas cedarwood	26.9	4.6	31.5	30.9	−1.9	26.9	4.5	31.4	29.7	−5.4
Geranium	28.0	2.0	30.0	33.6	12.0	28.0	19.2	47.2	46.4	−1.7
Cinnamon bark	27.0	3.2	30.2	26.0	−13.9	27.0	32.2	59.2	43.0	−27.4
Basil	35.0	50.2	85.2	44.0	−48.4	35.0	60.3	95.3	43.0	−54.9
Moroccan cedarwood	29.6	0.8	30.4	36.8	21.1	29.6	14.4	44.0	29.6	−32.7

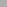
Light grey shading refers to synergistic co‐toxicity factors.

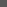
Dark grey shading refers to antagonistic co‐toxicity factors.

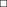
No shading corresponds to additive co‐toxicity factors.

### *Combinations with β‐cyfluthrin*

**Figure 4:**
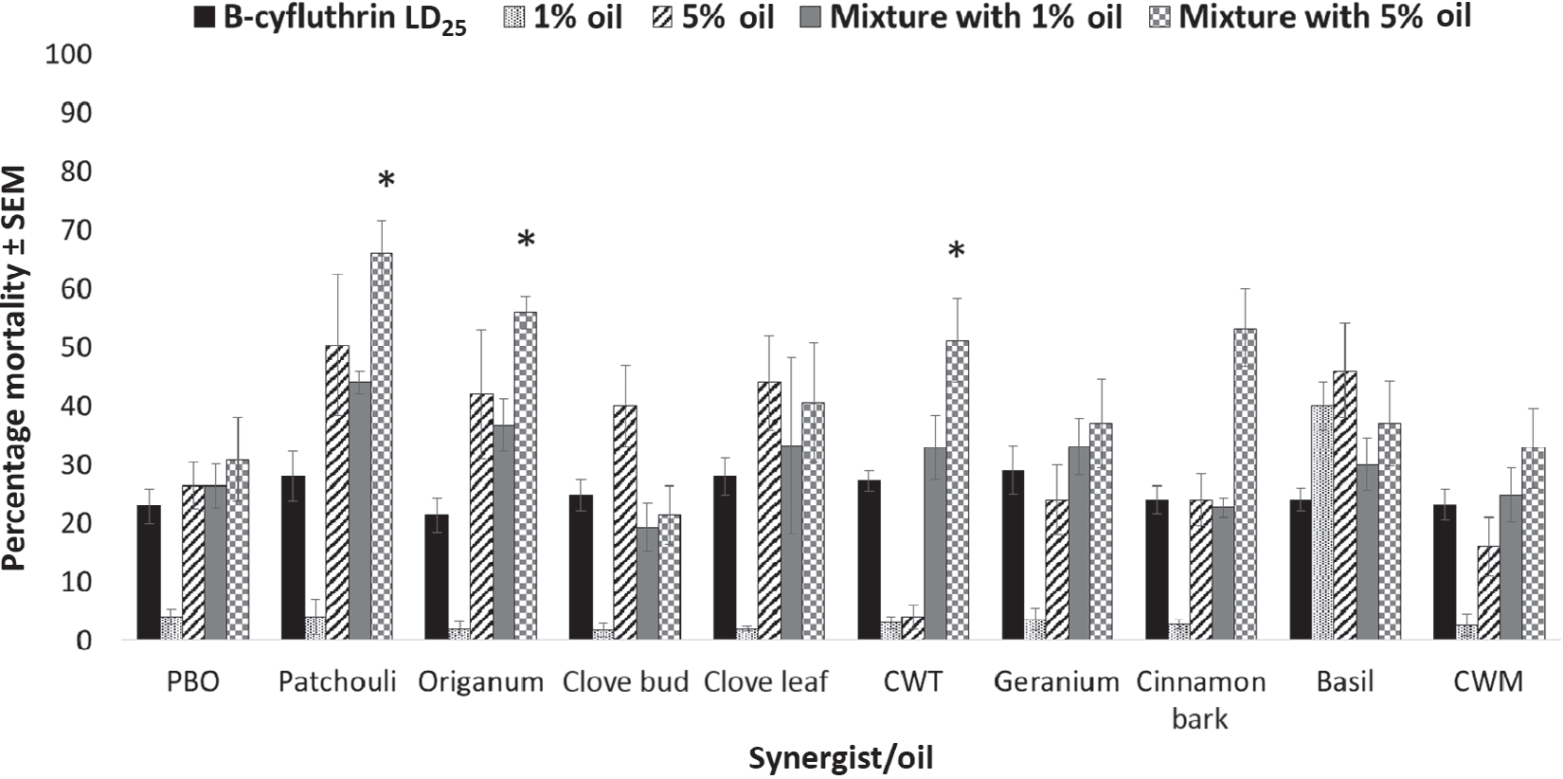
Percentage mortality in adult female *Aedes aegypti* at 24 h after application of each synergist or plant essential oil in combination with β‐cyfluthrin. *, statistically significant difference in percentage mortality caused by the 5% synergist/plant essential oil + LD_25_ β‐cyfluthrin combination compared with the LD_25_ β‐cyfluthrin‐alone application. PBO, piperonyl butoxide; CWT, Texas cedarwood; CWM, Moroccan cedarwood.

The increases in mortality caused by plant essential oils applied in combination with β‐cyfluthrin were considerable for select oils (Fig. 4). Two plant essential oils caused significant increases in *Ae. aegypti* mortality when applied in combination with the LD_25_ of β‐cyfluthrin at the 1% level. Both patchouli (*P* = 0.0138) and *Origanum* (*P* = 0.0165) oils caused significant enhancement compared with the LD_25_ of β‐cyfluthrin applied alone. Patchouli (*P* = 0.00155), *Origanum* (*P* = 0.0001), CWT (*P* = 0.012) and cinnamon bark (*P* = 0.0134) oils caused significant increases in mortality in *Ae. aegypti* compared with the LD_25_ of β‐cyfluthrin alone when applied in combination with the LD_25_ of β‐cyfluthrin at the 5% level. Mortality rates of 66.0 ± 6.0%, 56.0 ± 3.0%, 51.2 ± 7.0% and 53.3 ± 6.5% were observed in *Ae. aegypti* at 24 h after application of combinations of 5% patchouli, *Origanum*, CWT and cinnamon bark oils, respectively, and the LD_25_ of β‐cyfluthrin. Only the patchouli and *Origanum* oils were found to cause significant synergism of β‐cyfluthrin. Significant antagonism was noted for combinations of plant essential oils applied at the 5% level in combination with type II pyrethroids. Six oils were antagonistic in combinations with deltamethrin and seven oils were antagonistic in combination with β‐cyfluthrin at this level.

### *Combinations with malathion*

**Figure 5:**
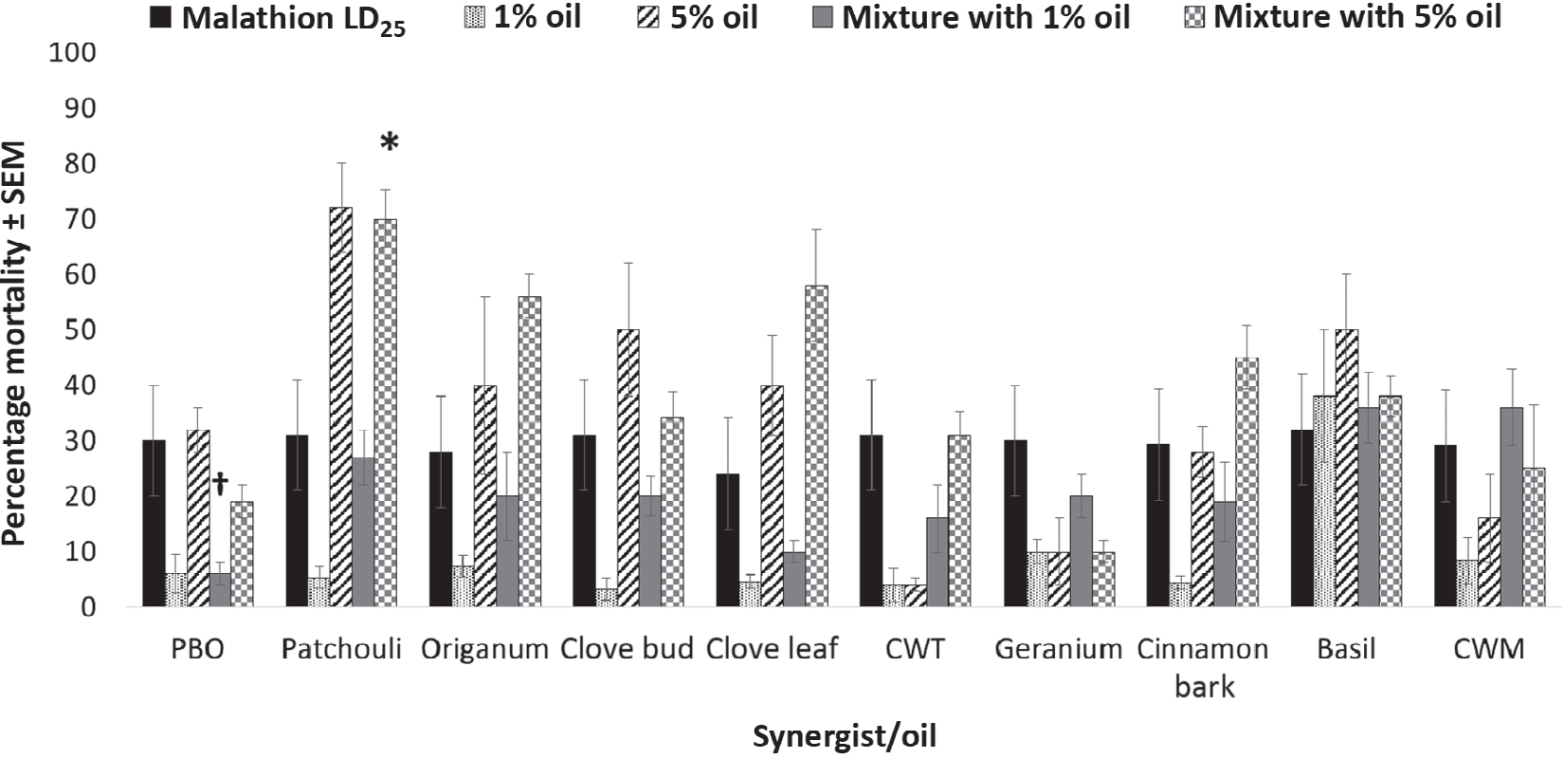
Percentage mortality in adult female *Aedes aegypti* at 24 h after application of each synergist or plant essential oil in combination with malathion. *, statistically significant difference in percentage mortality caused by the 5% synergist/plant essential oil + LD_25_ malathion combination compared with the LD_25_ malathion‐alone application. †, statistically significant difference in percentage mortality caused by the 1% synergist/plant essential oil + LD_25_ malathion combination compared with the LD_25_ malathion‐alone application. PBO, piperonyl butoxide; CWT, Texas cedarwood; CWM, Moroccan cedarwood.

The application of malathion in combination with varying concentrations of plant essential oils did not cause increases in *Ae. aegypti* mortality as significant as those achieved with the various combinations of plant essential oils and pyrethroids (Fig. 5). No oils caused increased mortality when applied at 1% in combination with the LD_25_ of malathion in comparison with malathion alone. However, PBO caused reduced mortality when applied at 1% in combination with malathion (*P* = 0.008). Only one oil, patchouli (*P* = 0.0004), caused a statistically significant increase in mortality when applied at 5% in combination with the LD_25_ of malathion, producing 70.0 ± 5.0% mortality at 24 h after application. Again, plant essential oil toxicity levels were similar to those observed with these oils in the previous pyrethroid combination studies (Figs 1, 2, 3, 4). Malathion was significantly antagonized by almost all plant essential oils, with all but one oil antagonizing malathion when applied to *Ae. aegypti* in combination with insecticide at both the 1% and 5% levels (Table 5). This antagonism was also caused by PBO, with co‐toxicity factors of − 83.3 and − 69.4 when applied at the 1% and 5% levels, respectively. In general, the levels of antagonism of plant essential oils were lower than that caused by PBO for both application concentrations. The one exception to this was geranium oil at the 5% level, which produced a co‐toxicity factor of – 75.0 compared with the − 69.4 caused by PBO. For the oils that did not produce antagonism, a purely additive co‐toxicity factor was calculated for CWM and clove leaf oils applied in combination with malathion at the 1% and 5% concentrations, respectively.

**Table 5:** Synergism and antagonism of an LD_25_ of β‐cyfluthrin by piperonyl butoxide (PBO) and select plant essential oils at various concentrations applied to *Aedes aegypti*. Values lower than − 20 suggest an antagonistic relationship, values between − 20 and 20 suggest an additive character, and values greater than 20 suggest a synergistic character.

	Oil/synergist 1%					Oil/synergist 5%				
	Mortality, %					Mortality, %				
Synergist/plant oil	β‐cyfluthrin	Synergist/oil alone	Expected	Observed	Co‐toxicity factor	β‐cyfluthrin	Synergist/oil alone	Expected	Observed	Co‐toxicity factor
PBO	22.9	4.0	26.9	26.3	−2.2	22.9	26.4	49.3	30.9	−37.3
Patchouli	28.0	4.0	32.0	44.0	37.5	28.0	50.4	78.4	66.0	−15.8
Origanum	21.3	2.0	23.3	36.7	57.5	21.3	42.0	63.3	56.0	−11.5
Clove bud	24.7	1.7	26.4	19.3	−26.9	24.7	40.0	64.7	21.3	−67.1
Clove leaf	28.0	2.0	30.0	33.2	10.7	28.0	44.0	72.0	40.6	−43.6
Texas cedarwood	27.2	3.0	30.2	32.8	8.6	27.2	4.0	31.2	51.2	64.1
Geranium	29.0	3.5	32.5	33.0	1.5	29.0	24.0	53.0	37.0	−30.2
Cinnamon bark	24.0	2.7	26.7	22.7	−15.0	24.0	24.0	48.0	53.3	11.0
Basil	24.0	40.0	64.0	30.0	−53.1	24.0	46.0	70.0	37.0	−47.1
Moroccan cedarwood	23.2	2.6	25.8	24.8	−3.9	23.2	16.0	39.2	32.8	−16.3

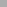
Light grey shading refers to synergistic co‐toxicity factors.

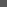
Dark grey shading refers to antagonistic co‐toxicity factors.

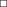
No shading corresponds to additive co‐toxicity factors.

### *Percentage enhancement*

**Figure 6:**
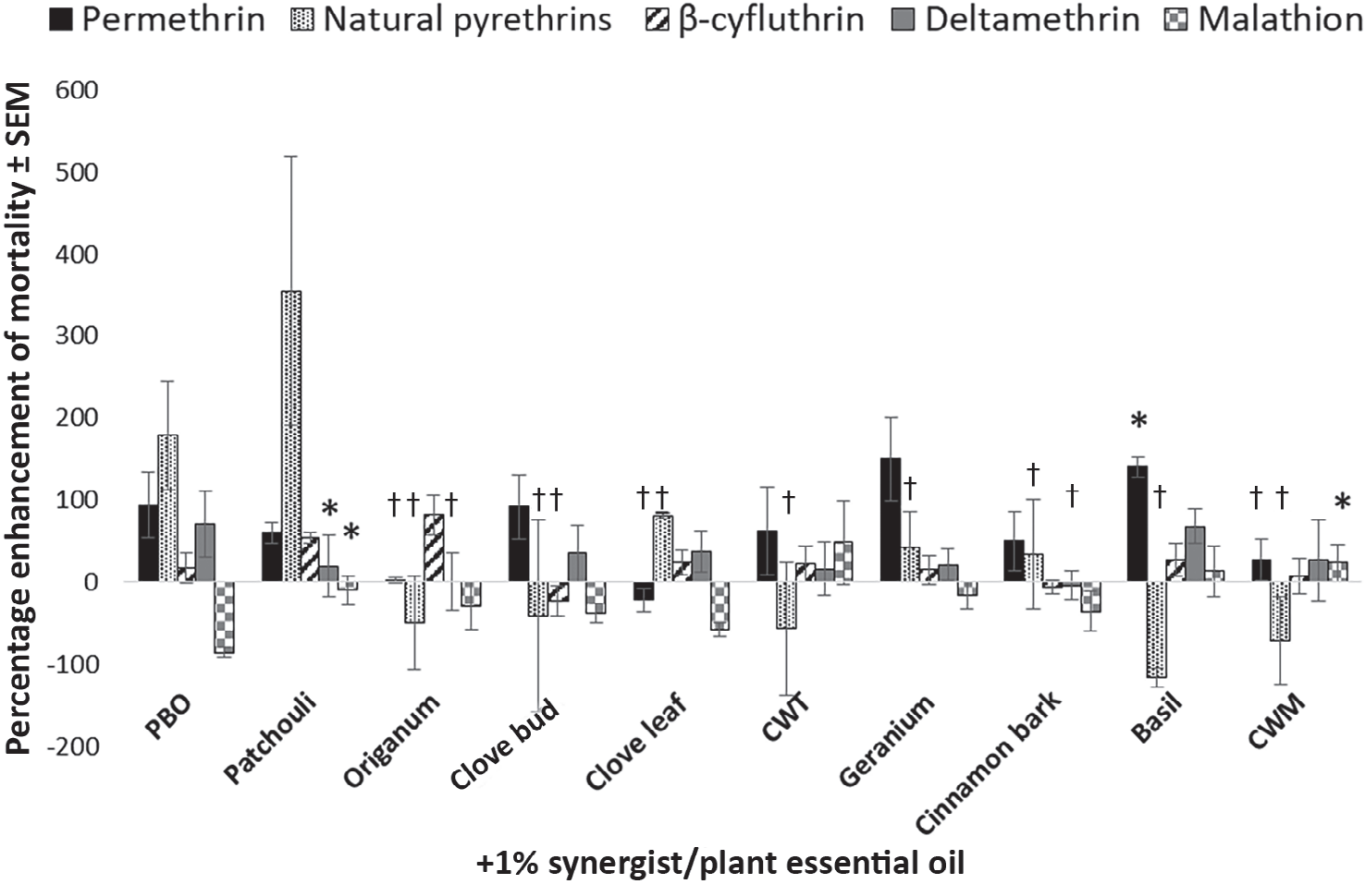
Percentage enhancement of mortality ± standard error of the mean (SEM) in adult female *Aedes aegypti* at 24 h post‐application of a mixture of 1% synergist or plant essential oil compared with the LD_25_ of insecticide (synthetic insecticide or natural pyrethrins) alone. Each bar represents the average percentage enhancement for 1% plant essential oil in combination with the LD_25_ of each insecticide. The average percentage enhancement for each mixture was compared with the percentage enhancement caused by 1% piperonyl butoxide (PBO) + each insecticide via a one‐way anova with a Student–Newman–Kuel test (α = 0.05). *, oils that produced statistically significant increases in percentage enhancement compared with PBO; †, oils that produced statistically significant decreases in percentage enhancement compared with PBO. CWT, Texas cedarwood; CWM, Moroccan cedarwood.

**Figure 7:**
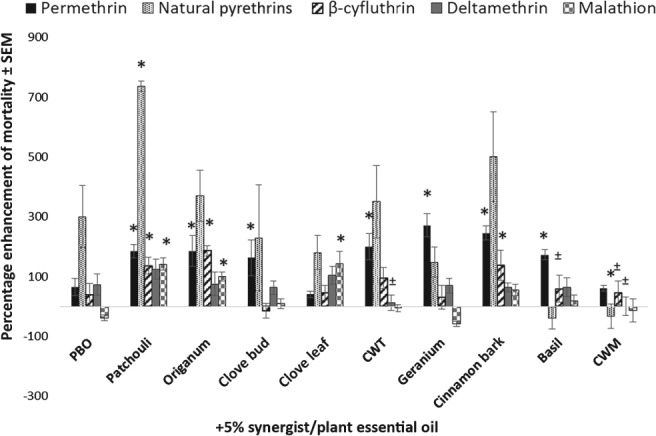
Percentage enhancement of mortality ± standard error of the mean (SEM) in adult female *Aedes aegypti* at 24 h post‐application of a mixture of 5% synergist or plant essential oil compared with the LD_25_ of insecticide (synthetic insecticide or natural pyrethrins) alone. Each bar represents the average percentage enhancement for 5% plant essential oil in combination with the LD_25_ of each insecticide listed above. The average percentage enhancement for each mixture was compared with the percentage enhancement caused by 5% piperonyl butoxide (PBO) + each insecticide via a one‐way anova with a Student–Newman–Kuel test (α = 0.05). *, statistically significant percentage enhancement (increased or decreased enhancement). CWT, Texas cedarwood; CWM, Moroccan cedarwood.

Another metric, percentage enhancement, was used to evaluate the degree to which plant essential oils increased the toxicity of pyrethroids or malathion when applied to *Ae. aegypti* in combination at 1% (Fig. 6) or 5% (Fig. 7). At the 1% level, PBO positively enhanced the effects of all the pyrethroids tested in *Ae. aegypti* by 93.5 ± 40.0%, 178.6 ± 66.0%, 70.2 ± 40.0% and 16.7 ± 19.0% when applied in combination with permethrin, natural pyrethrins, deltamethrin and β‐cyfluthrin, respectively. Piperonyl butoxide caused a negative enhancement when applied at 1% in combination with malathion (− 86.0 ± 6.0%). The majority of the oils screened performed similarly to PBO when applied at the 1% concentration with the respective insecticide. The exceptions to this were patchouli (*P* = 0.001) and CWM (*P* = 0.002) oils, which caused statistically higher percentage enhancements of malathion than PBO. A number of oils produced statistically lower levels of enhancement than PBO when applied in specific combinations with various insecticides. These included *Origanum* (*P* = 0.0009), clove leaf (*P* = 0.042), CWM (*P* = 0.0308) oils when applied in combination with permethrin; all oils except patchouli (*P* < 0.05) oil in combination with natural pyrethrins; *Origanum* (*P* = 0.0461) and cinnamon bark (*P* = 0.0249) oils in combination with deltamethrin, and patchouli (*P* = 0.0173), *Origanum* (*P* = 0.0049) and clove bud (*P* = 0.0252) oils in combination with β‐cyfluthrin. At the 5% level, many plant essential oils significantly outperformed PBO. For example, every plant essential oil outperformed PBO in combination with at least one insecticide, with the exception of clove leaf oil. Some oils, such as patchouli oil, outperformed PBO in combination with three of the four pyrethroids screened. Summaries of the *P*‐values for oil–insecticide combinations that produced statistically higher enhancement values compared with PBO and the corresponding insecticide are provided in Appendix S1.

## Discussion

The LD_50_ and LD_25_ values of various insecticides applied topically to adult female *Ae. aegypti* were successfully determined in this study in order to later evaluate the synergistic potentials of plant essential oils compared with that of PBO. The LD_50_ values obtained in this study for each insecticide were similar to those reported in the literature (Agramonte *et al*. 2017; Norris *et al*. 2015). In general, no insecticide LD_50_ differed from values reported in the literature by greater than one order of magnitude. More than 700 mosquitoes were used to calculate a robust LD_25_ value for each insecticide to be utilized in subsequent experiments. Table 1 highlights the dose–response statistics for each insecticide utilized in this study. Of the insecticides screened initially, β‐cyfluthrin was the most toxic insecticide applied to mosquitoes. This was followed by deltamethrin, permethrin, malathion and natural pyrethrins. Type II pyrethroids were significantly more toxic than type I pyrethroids in terms of LD_25_ and LD_50_ values. For each insecticide screened, the probit model sufficiently described the dose–response data for each insecticide to be used in the subsequent synergism explorations. Heterogeneity co‐factors were applied to the models as χ^2^‐values were high, but this is most likely to be attributable to the large *n*‐value used for each insecticide.

A panel of pyrethroids in combination with either PBO, a synergist that is currently used to enhance the toxicity of various insecticidal ingredients for the control of mosquitoes, or plant essential oils was screened. In the present study, PBO did not cause significant increases in type I pyrethroid mortality in *Ae. aegypti* at the 1% rate and produced increases in mortality effected by natural pyrethrins only at the 5% application concentration. This may be attributable to the application protocol utilized in this study or to the concentration at which PBO was applied. Numerous studies have shown that the efficacy of PBO as a synergist is dependent on the concentration at which it is applied (Hewlett, 1969; Guglielmone *et al*., 1999; Dehkordi *et al*., 2017; Nikpour *et al*., 2017). This is further evidenced by the inability of PBO to increase the toxicity of both type II pyrethroids screened in this study (deltamethrin and β‐cyfluthrin). It is possible that the application concentrations in this study were too low for PBO to be effective as a synergist for type I and II pyrethroids. By contrast, numerous plant essential oils were capable of increasing the efficacy of both type I and II pyrethroids at these application concentrations. A majority of plant oils produced significant increases in the mortality rates achieved by both permethrin and natural pyrethrins, and many oils produced a higher percentage mortality than PBO. Plant essential oils were less likely to increase the toxicity of type II pyrethroids than that of type I pyrethroids. Among the oils that most significantly increased the toxicity of type II pyrethroids, patchouli oil was the only one to increase the toxicity of both deltamethrin and β‐cyfluthrin. This may reflect its intrinsic toxicity as it was noted to be one of the most toxic plant essential oils screened in a previous study (Norris *et al*., 2015). Other oils that caused significant increases in type II pyrethroid toxicity were *Origanum*, cinnamon bark, CWT and geranium oils. Of these oils, cinnamon bark and *Origanum* oils were also noted to be among the most toxic plant essential oils screened previously against *Ae. aegypti* adults (Norris *et al*., 2015). Texan cedarwood and geranium oils may contribute to the toxicity of deltamethrin and β‐cyfluthrin, respectively, by synergistic interactions as they were not among the most toxic oils (Norris *et al*., 2015).

However, PBO applied in combination with an LD_25_ of malathion at a concentration of 1% caused a significant decrease in 24‐h mortality compared with the LD_25_ of malathion applied alone. A lower percentage mortality of malathion in combination with PBO may be produced by the inhibition of oxygenases, which are required to activate this pro‐insecticide into malaoxon (Hoffman *et al*., 1954; Rai & Roan, 1959; Forsyth & Chambers, 1989; Dos Santos *et al*., 2010). A majority of oils either decreased the efficacy of malathion or produced no statistically significant increase in the 24‐h mortality produced by malathion. This may reflect the inhibition of detoxification enzymes by plant essential oils, which limits the toxicity of malathion in a similar manner to the effect observed with combinations of this insecticide and PBO. At the 5% level, only patchouli oil caused a statistically significant increase in mortality in *Ae. aegypti* when applied in combination with an LD_25_ of malathion. This increase in toxicity may have been caused by the intrinsic toxicity of patchouli oil as was observed when this oil was combined with pyrethroids.

Enhancement levels (as percentages) caused by PBO or plant essential oils were also characterized for each of the insecticides. Establishing the percentage enhancement is another method of quantifying the increased efficacy of insecticides applied in combination with plant essential oils, and accounts for both additive and synergistic interactions between agents within a mixture (Gross *et al*. 2017a). Using this metric, it is apparent that plant essential oils significantly enhance the effects of a wide variety of insecticides to a higher degree than PBO in many cases. In general, percentage enhancement was more pronounced for plant essential oils applied at the 5% level in combination with various pyrethroids. This may be attributable to their intrinsic toxicity at the 5% application concentration. In addition, enhancement occurred more frequently for type I pyrethroids applied in combination with plant essential oils compared with type II pyrethroids. From an adult mosquito control perspective, this metric may be the most informative as increased toxicity may be the most practical and desirable endpoint.

Finally, to explore whether plant essential oils synergize these insecticides, the co‐toxicity factor was used as a means of assessing the synergistic potentials of these various oils when applied in mixtures with various insecticides. Tables 2, 3, 4, 5, 6 highlight the various co‐toxicity factors of each insecticide screened with plant essential oils and insecticides or plant essential oils applied alone at the LD_25_ level or the 1% or 5% levels, respectively. As discussed in previous work, co‐toxicity factors greater than 20 indicate a synergistic interaction between two toxicants present in an insecticidal mixture, values between 20 and − 20 indicate a purely additive interaction, and values below − 20 suggest an antagonistic interaction (Mansour *et al*., 1966). From these data, it is evident that many plant essential oils synergize diverse insecticides for *Ae. aegypti*. Piperonyl butoxide synergized the toxicity of both permethrin and natural pyrethrins at the 1% but not the 5% concentration. At the 5% concentration, PBO acted more antagonistically with co‐toxicity factors of − 24.46 and − 14.1 for permethrin and natural pyrethrins, respectively. Interestingly, PBO did not synergize type II pyrethroids in this study (deltamethrin and β‐cyfluthrin) at either the 1% or 5% application level when used in combination with these insecticides in *Ae. aegypti*. In general, plant essential oils were also less likely to synergize the type II pyrethroids used in this study; however, a number of oils caused synergism for select type II pyrethroids. This highlights a potential advantage of utilizing plant essential oils rather than PBO in future insecticidal formulations. As resistance to deltamethrin and other type II pyrethroids builds, plant essential oils may represent promising avenues for the development of new insecticides. Esterase‐ and oxygenase‐mediated clearance of pyrethroids is less pronounced for type II pyrethroids in general (Craig *et al*., 1957). The lesser potential of plant essential oils to synergize type II pyrethroids may reflect the lower levels of enzymatic clearance or detoxification of these insecticides. This difference in type I vs. type II pyrethroid metabolism is further evidenced by the differential synergism caused by PBO applied in combination with either a type I or type II pyrethroid. In previous work, PBO synergized natural pyrethrins (a type I pyrethroid) almost 300‐fold, whereas it enhanced deltamethrin (a type II pyrethroid) only 10‐fold (Soderlund & Casida, 1977; Casida *et al*., 1983). In the present study, lower levels of type II pyrethroid synergism caused by PBO and plant oils were evident. Piperonyl butoxide and a majority of plant essential oils caused significant antagonism when applied in combination with malathion at both the 1% and 5% concentrations. The antagonism of this insecticide by plant essential oils in a similar manner to PBO further indicates that plant essential oils may act to inhibit oxidative processes important for the activation of this pro‐insecticide.

**Table 6:** Antagonism of an LD_25_ of malathion by piperonyl butoxide (PBO) and select plant essential oils at various concentrations applied to *Aedes aegypti*. Values lower than − 20 suggest an antagonistic relationship, values between − 20 and 20 suggest an additive character, and values greater than 20 suggest a synergistic character.

	Oil/synergist 1%					Oil/synergist 5%				
	Mortality, %					Mortality, %				
Synergist/plant oil	Malathion	Synergist/oil alone	Expected	Observed	Co‐toxicity factor	Malathion	Synergist/oil alone	Expected	Observed	Co‐toxicity factor
PBO	30.0	6.0	36.0	6.0	−83.3	30.0	32.0	62.0	19.0	−69.4
Patchouli	31.0	5.3	36.3	27.0	−25.6	31.0	72.0	103.0	70.0	−32.0
Origanum	28.0	7.4	35.4	20.0	−43.5	28.0	40.0	68.0	56.0	−17.6
Clove bud	31.0	3.2	34.2	20.0	−41.5	31.0	50.0	81.0	34.0	−58.0
Clove leaf	24.0	4.6	28.6	10.0	−65.0	24.0	40.0	64.0	58.0	−9.4
Texas cedarwood	31.0	4.0	35.0	16.0	−54.3	31.0	4.0	35.0	26.0	−25.7
Geranium	30.0	10.0	40.0	20.0	−50.0	30.0	10.0	40.0	10.0	−75.0
Cinnamon bark	29.3	4.4	33.7	18.7	−44.5	29.3	28.0	57.3	45.3	−20.9
Basil	32.0	38.0	70.0	36.0	−8.6	32.0	50.0	82.0	38.0	−53.7
Moroccan cedarwood	29.0	8.4	37.4	36.0	−3.7	29.0	16.0	45.0	25.0	−44.4

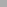
Light grey shading refers to synergistic co‐toxicity factors.

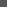
Dark grey shading refers to antagonistic co‐toxicity factors.

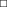
No shading corresponds to additive co‐toxicity factors.

Multiple oils enhanced the toxicity of diverse pyrethroids in this study. Of these oils, patchouli was the most successful as it synergized permethrin, natural pyrethrins and β‐cyfluthrin at the 1% concentration. Numerous other plant essential oils also successfully synergized various pyrethroids at the 5% concentration. Of the oils, CWT and geranium oils were the most successful synergists against multiple types of pyrethroid. When applied at the 5% concentration in combination with various pyrethroids, CWT oil synergized three of the four pyrethroids screened; only deltamethrin was not synergized by this plant essential oil. Moreover, this oil consistently showed the highest co‐toxicity factor of all the plant essential oils screened. Finally, all plant essential oils screened caused significant antagonism of malathion when applied in combination with the LD_25_ of malathion. This was observed when plant essential oils were combined with malathion at both the 1% and 5% concentrations. This result may indicate that particular terpenoids in CWT and geranium oils act to interfere with detoxification processes, leading to higher bioavailability of the topically applied pyrethroid. This in turn could lead to the higher percentage mortality and account for the synergism seen in this study.

Multiple other studies have suggested that plant essential oils enhance the toxicity of various permethrin and natural pyrethrins (Joffe *et al*., 2012; Gross *et al*., 2017b). The mechanism of this enhancement has not been fully explored, but it has been suggested that various components within plant essential oils prevent the detoxification of pyrethroids and other insecticides. Previous studies demonstrated that select plant essential oil terpenoids and plant extracts significantly inhibited detoxification enzymes (Waliwitiya *et al*., 2012; Carreño Otero *et al*., 2018) and hence it is possible that the terpenoids in the plant essential oils screened in this study work in a similar manner. The present study further confirms this finding with whole organism toxicological endpoints, such as 24‐h mortality and co‐toxicity factors. The observation of the antagonism of malathion further suggests that plant essential oils act to inhibit detoxification processes, specifically monooxygenases, in exposed *Ae. aegypti*. As malathion requires activation by monooxygenases, the observed antagonism of malathion strongly supports the premise that these activation processes are inhibited or downregulated. Although promising synergistic plant oils were identified, no plant essential oil caused significant synergism of all pyrethroids and antagonism of malathion. Instead, the ability of plant essential oils to act as synergists depends on the concentrations at which they are applied and the insecticide with which they are applied. Pyrethroids are selectively degraded by a number of specific detoxification enzymes within insects (Joffe *et al*., 2012; Gross *et al*. 2017a). The data presented strongly suggest that particular plant terpenoids have higher levels of propensity for interfering with select enzyme systems than others. This may account for the variably synergistic profiles of the various plant essential oils, which appeared to be highly specific to the synthetic insecticide chosen for combined application. Although these results provide promising leads for the use of plant essential oils to synergize pyrethroids in formulation, further work must be completed to fully implicate plant essential oils as inhibitors of various detoxification processes. Moreover, the characterization of this synergism in other insect models of agricultural, urban or veterinary relevance has yet to be explored and would certainly elucidate the potential of this approach to pest control in general.

Plant essential oils may represent a more environmentally responsible means of increasing the efficacy of current insecticidal formulations that are beginning to lose efficacy in the field as a result of insecticide resistance. Plant essential oils and natural compounds are generally safer for humans and non‐target organisms and are less persistent in the environment than many conventional insecticides (Isman, 2000). This study demonstrates that plant essential oils have the ability to increase the toxicity of various pyrethroids by synergizing these insecticides. Plant essential oils could be included in spatial sprays and in indoor residual spraying campaigns, but further work is required to better understand how these chemistries should be formulated into future control products and technologies, and how these formulations act in other mosquito strains. Pest control campaigns must begin to consider the development of more sustainable and eco‐friendly insecticidal formulations. Given the lengthy process involved in the development of novel insecticides, the use of novel, safe synergists as an alternative means of increasing insecticide efficacy may be both viable and responsible.

## Supporting information


**Appendix S1.** *P*‐values for comparisons of enhancement percentages (oils with piperonyl butoxide).

**Table S1.** Gas chromatography–mass spectrometry data for the oils included in this study.
