# Supplementary material for: Plant essential oils synergize various pyrethroid insecticides and antagonize malathion in Aedes aegypti
Source: Med Vet Entomol. 2019 May 17;33(4):453–66. doi: 10.1111/mve.12380 (PMC6899815; doi:10.1111/mve.12380)
Supplement: Supplementary file 1 — Appendix S1. P‐values for comparisons of enhancement percentages (oils with piperonyl butoxide). [file MVE-33-453-s001.docx]

**S1. P-values for comparison of enhancement percentages (oils to PBO)**

| oil/synergist applied at 1% | p-values for combinations with various insecticides | | | | |
| --- | --- | --- | --- | --- | --- |
|  | permethrin | deltamethrin | β-cyfluthrin | natural pyrethrins | malathion |
| Patchouli | 0.112 | 0.097 | **0.0173** | 0.0688 | **0.002** |
| Origanum | **0.0009** | **0.0461** | **0.0049** | **0.0026** | **0.028** |
| Clove Bud | 0.95 | 0.247 | **0.0252** | **0.0132** | **0.0036** |
| Clove Leaf | **0.0003** | 0.2417 | 0.61 | **0.0461** | **0.0097** |
| CWT | 0.323 | 0.0945 | 0.729 | **0.0039** | **0.0109** |
| Geranium | 0.0871 | 0.0995 | 0.8909 | **0.0198** | **0.0026** |
| Cinnamon Bark | 0.108 | **0.0249** | 0.0934 | **0.0237** | **0.0251** |
| Basil | **0.0375** | 0.9338 | 0.474 | **0.0003** | **0.0059** |
| CWM | **0.0308** | 0.2166 | 0.54 | **0.0015** | **0.0014** |
|  |  |  |  |  |  |
|  |  |  |  |  |  |
|  |  |  |  |  |  |
| oil synergist applied at 5% | p-values for combinations with various insecticides | | | | |
|  | permethrin | deltamethrin | β-cyfluthrin | natural pyrethrins | malathion |
| Patchouli | **0.0049** | 0.0875 | **0.0079** | **0.0004** | **0.0001** |
| Origanum | **0.0048** | 0.923 | **0.0007** | 0.366 | **0.0001** |
| Clove Bud | **0.0203** | 0.421 | 0.07 | 0.493 | **0.0018** |
| Clove Leaf | 0.212 | 0.703 | 0.809 | 0.121 | **0.0001** |
| CWT | **0.0018** | **0.0421** | 0.0839 | 0.555 | **0.0051** |
| Geranium | **0.0001** | 0.915 | 0.74 | 0.0587 | **0.0343** |
| Cinnamon Bark | **0.0001** | 0.717 | **0.0176** | 0.1793 | **0.0001** |
| Basil | **0.0012** | 0.747 | 0.515 | **0.0018** | **0.001** |
| CWM | 0.7882 | **0.0269** | 0.848 | **0.0021** | **0.2015** |
