# Supplementary material for: Plant essential oils synergize various pyrethroid insecticides and antagonize malathion in Aedes aegypti
Source: Med Vet Entomol. 2019 May 17;33(4):453–66. doi: 10.1111/mve.12380 (PMC6899815; doi:10.1111/mve.12380)
Supplement: Supplementary file 2 — Table S1. Gas chromatography–mass spectrometry data for the oils included in this study. [file MVE-33-453-s002.docx]

| **Basil oil Egyptian Type** |  |
| --- | --- |
| Compounds | Peak Area % |
| β-pinene | 1.49 |
| 1,8-cineole | 9.9 |
| linalool | 42.92 |
| α-terpineol | 0.51 |
| estragole | 1.1 |
| bornyl acetate | 2.17 |
| eugenol | 5.63 |
| β-elemene | 4.24 |
| trans-α-bergamotene | 6.36 |
| α-caryophyllene | 1.92 |
| β-copaene | 4 |
| bicyclogermacrene | 1.12 |
| α-bulnesene | 0.74 |
| δ-cadinene | 4.09 |
| unknown | 2.24 |
| unknown | 4.4 |
| unknown | 3.07 |
| unknown | 4.1 |
|  |  |
| **Cedarwood oil Texas Type** | |
| Compounds | Peak Area % |
| α-cedrene | 13.33 |
| β-cedrene | 3.98 |
| thujopsene | 46.34 |
| cuparene | 4.05 |
| widdrol | 1.81 |
| cedrol | 30.49 |
|  |  |
| Cedarwood oil Moroccan Type | |
| Compounds | Peak Area % |
| limona ketone | 0.78 |
| sibirene + longifolene (50/50) | 0.79 |
| α-himachalene | 20.82 |
| γ-himachalene | 14.76 |
| β-himachalene | 57.88 |
| deodarone | 1.98 |
| trans-α-alantone | 2.99 |
|  |  |
| Cinnamon Bark oil |  |
| Compounds | Peak Area % |
| para-cymene | 1.03 |
| β-phellandrene | 1.84 |
| linalool | 3.07 |
| trans-cinnamaldehyde | 82.43 |
| eugenol | 1.86 |
| β-caryophyllene | 6.62 |
| cinnamyl acetate | 3.15 |
|  |  |
| Clove bud oil |  |
| Compounds | Peak Area % |
| eugenol | 79.31 |
| β-caryophyllene | 7.7 |
| α-caryophyllene | 1.06 |
| eugenyl acetate | 11.93 |
|  |  |
| Geranium oil Bourbon Type | |
| Compounds | Peak Area % |
| linalool | 2.58 |
| isomenthone + menthone (80/20) | 10.42 |
| citronellol | 48.49 |
| geraniol | 4.59 |
| citronellyl formate | 15.62 |
| 10-eip-γ-eudesmol | 18.3 |
|  |  |
| Clove leaf oil |  |
| Compounds | Peak Area % |
| eugenol | 81.69 |
| β-caryophyllene | 16.32 |
| α-caryophyllene | 1.99 |
|  |  |
|  |  |
| Origanum oil |  |
| Compounds | Peak Area % |
| α-pinene | 1.38 |
| β-pinene | 1.15 |
| α-terpinene | 1.19 |
| para-cymene | 12.01 |
| γ-terpinene | 8.92 |
| linalool | 1.47 |
| carvacrol + thymol (95/5) | 72.41 |
| β-caryophyllene | 1.46 |
|  |  |
| Patchouli oil |  |
| Compounds | Peak Area % |
| β-patchoulene | 3.44 |
| β-caryophyllene | 3.57 |
| α-guaiene | 15.3 |
| seychellene | 8.56 |
| α-patchoulene | 5.49 |
| γ-patchoulene | 3.17 |
| aciphyllene | 3.06 |
| α-bulnesene | 19.09 |
| β-selinene | 1.87 |
| patchouli alcohol | 36.45 |
|  |  |
|  |  |
|  |  |
|  |  |
|  |  |
|  |  |
|  |  |
|  |  |
|  |  |
|  |  |
|  |  |
|  |  |
|  |  |
|  |  |
|  |  |
|  |  |
|  |  |
|  |  |
|  |  |
|  |  |
|  |  |
|  |  |
|  |  |
|  |  |
|  |  |
|  |  |
|  |  |
|  |  |
|  |  |
|  |  |
|  |  |
|  |  |
|  |  |
|  |  |
|  |  |
|  |  |
